# Supplementary material for: Asynchronous magnetic resonance elastography: Shear wave speed reconstruction using noise correlation of incoherent waves
Source: Magn Reson Med. 2022 Oct 27;89(3):990–1001. doi: 10.1002/mrm.29502 (PMC9792433; doi:10.1002/mrm.29502)
Supplement: Supplementary file 1 — DATA S1 MATLAB code used to generate the numerical simulations of Figure 1 [file MRM-89-990-s002.zip › k-Wave/helpfiles/example_pr_3D_tr_spherical_sensor.html]

3D Time Reversal Reconstruction For A Spherical Sensor Example (k-Wave)


# 3D Time Reversal Reconstruction For A Spherical Sensor Example

This example demonstrates the use of k-Wave for the time-reversal reconstruction of a three-dimensional photoacoustic wave-field recorded over a spherical sensor.
The sensor data is simulated and then time-reversed using `kspaceFirstOrder3D`.
It builds on the 2D Time Reversal Reconstruction For A Circular Sensor and 3D Time Reversal Reconstruction For A Planar Sensor examples.

- Open the file in the MATLAB Editor
- Run the file in MATLAB

## Contents

- Simulating the sensor data
- Running the reconstruction
- Interpolating incomplete sensor data

## Simulating the sensor data

The sensor data is simulated using `kspaceFirstOrder3D` with an initial pressure distribution created using `makeBall`.
A Cartesian sensor mask of 100 points evenly distributed around a sphere (using the Golden Section Spiral method) is created using `makeCartSphere`.
A visualisation of the initial pressure and the sensor mask using `cart2grid` and `voxelPlot` is shown below.


## Running the reconstruction

A plot of the initial pressure distribution and the reconstructed distribution using time-reversal are shown below (the three displayed image planes intersect at the centre of ball shown above). The plot scale for the reconstructed image has the plot limits reduced by a factor of 20.


## Interpolating incomplete sensor data

The reconstruction can again be improved by interpolating the sensor data onto a continuous sensor surface. This is achieved using `interpCartData` and a binary sensor mask of a continuous sphere (created here using `makeSphere`) that is spatially equivalent to the Cartesian measurement grid. This function calculates the equivalent time-series at the sensor positions on the binary sensor mask from those on the Cartesian sensor mask via interpolation (nearest neighbour is used by default).

```
% create a binary sensor mask of an equivalent continuous sphere
pixel_radius = round(sensor_radius / kgrid_recon.dx);
binary_sensor_mask = makeSphere(kgrid_recon.Nx, kgrid_recon.Ny, kgrid_recon.Nz, pixel_radius);

% interpolate data to remove the gaps and assign to time reversal data
sensor.time_reversal_boundary_data = interpCartData(kgrid_recon, sensor_data, sensor_mask, binary_sensor_mask);
```

Details of the interpolation are printed to the command line.

```
Interpolating Cartesian sensor data...
  interpolation mode: nearest
  number of Cartesian sensor points: 100
  number of binary sensor points: 4234
  computation completed in 0.02934s
```

The reconstruction after interpolation is shown below. The plot limits are set to match those of the initial pressure plot shown above.
